# Induction of autophagy improves embryo viability in cloned mouse embryos

**DOI:** 10.1038/srep17829

**Published:** 2015-12-08

**Authors:** XingHui Shen, Na Zhang, ZhenDong Wang, GuangYu Bai, Zhong Zheng, YanLi Gu, YanShuang Wu, Hui Liu, DongJie Zhou, Lei Lei

**Affiliations:** 1 Department of Histology and Embryology, Harbin Medical University, Harbin, China

## Abstract

Autophagy is an essential cellular mechanism that degrades cytoplasmic proteins and organelles to recycle their components. Moreover, autophagy is essential for preimplantation development in mammals. Here we show that autophagy is also important for reprogramming in somatic cell nuclear transfer (SCNT). Our data indicate that unlike fertilized oocytes, autophagy is not triggered in SCNT embryos during 6 hours of activation. Mechanistically, the inhibited autophagic induction during SCNT activation is due to the cytochalasin B (CB) caused depolymerization of actin filaments. In this study, we induced autophagy during SCNT activation by rapamycin and pp242, which could restore the expected level of autophagy and significantly enhance the development of SCNT embryos to the blastocyst stage when compared with the control (68.5% and 68.7% vs. 41.5%, *P* < 0.05). Furthermore, the treatment of rapamycin and pp242 accelerates active DNA demethylation indicated by the conversion of 5 mC to 5 hmC, and treatment of rapamycin improves degradation of maternal mRNA as well. Thus, our findings reveal that autophagy is important for development of SCNT embryos and inhibited autophagic induction during SCNT activation might be one of the serious causes of low efficiency of SCNT.

The oocyte, one of the most highly differentiated cells, suddenly changes into a highly undifferentiated state after fertilization. This ‘reprogramming’ occurs both in the nucleus and the cytoplasm. During this process, maternal and paternal genomes are epigenetically modified, pluripotency genes are expressed, and inherited maternal proteins are erased^1^,^2^. The physiological role of autophagy in zygote reprogramming has been investigated intensely in recent years and has also provided insight into the underlying molecular signaling events. Maternal mRNA and proteins are rapidly degraded after the two-cell stage in the embryos, and new mRNA and proteins encoded by the zygotic genome are synthesized, leading to marked changes in the protein species synthesized after the four-cell to eight-cell stages. Moreover, the degradation of maternal proteins and RNAs may be necessary for the activation of the zygotic genome^3^. The ubiquitin–proteasome system is essential for the degradation of short-lived proteins during zygote reprogramming, whereas long-lived proteins and organelles are removed by autophagy^1^,^4^.

Macroautophagy (hereafter referred as autophagy in this report) is one of the basic processes of degrading unnecessary or dysfunctional cell components^5^,^6^. This process begins with the engulfment of the targeted components including macromolecules (proteins, glycogens, lipids and nucleotides, etc.) and organelles (e.g. mitochondria, peroxisomes and endoplasmic reticulum) in double-membrane bound autophagosomes. Once autophagosomes are formed, their outer membranes will fuse with lysosomes, with consequent disintegration of the inner autophagosomal membranes and degradation of the contents of autophagosomes by lysosomal enzymes. The produced catabolites including amino acids AA and free fatty acids FFA are rapidly made available in the cytoplasm for recycling. Thus, autophagy provides a “recycling system”, and this system plays a key role in various physiological processes such as adaptation to starvation, quality control of cytoplasmic constituents, and clearance of intracellular pathogens^7^,^8^,^9^.

The earliest autophagic event in mammalian development is observed in fertilized oocytes. The Atg5-deficient oocytes fail to develop beyond the four-to eight-cell stage after fertilization with Atg5^−/−^ sperms^4^. Furthermore, absence of autophagy impairs the embryonal capacity for protein neo-synthesis, a consequence that likely arises from the lack of maternal protein removal and the associated incapacity to recycle amino acids^4^. Therefore, the autophagy may also be required for the active elimination of unnecessary proteins and organelles that accumulate within oocytes or to facilitate reprogramming by degrading maternal suppressors of the zygotic gene program. The precise role of autophagy during this process is not fully understood. Because the rate of protein synthesis is reduced in autophagy-defective embryos, normal levels of autophagy may be necessary for the production of sufficient amino acids for protein synthesis. Given that autophagy is an intracellular recycling system, these different possibilities are not mutually exclusive.

Recently, autophagy has also been shown to participate in the regulation of the somatic reprogramming process. Reprogramming of somatic nuclei into a pluripotent state can be achieved through either ectopic expression of reprogramming factors in somatic cells to generate induced pluripotent stem cells (iPSCs)^10^ or somatic cell nuclear transfer (SCNT)^11^. Somatic cell reprogramming involves epigenetic modification, changes in gene expression, protein degradation, and protein synthesis. Pharmacological induction of autophagy increases the reprogramming efficiency of mouse embryonic fibroblasts (MEF) to iPSCs^12^. In addition, sox2 initiates autophagy by repressing mTOR expression early in reprogramming and sox2-dependent temporal regulation of autophagy is a key step in cellular reprogramming processes during iPSC generation^13^. These findings suggest the intriguing possibility that autophagy could serve as a positive regulator of induced pluripotency. Autophagy might promote the induction of pluripotency by counteracting cellular senescence and apoptosis; both are thought to be barriers to reprogramming^14^.

Although several reports discovered the autophagic function as facilitate reprogramming in fertilized embryos and process of iPSC generation, the role of autophagy in SCNT is still poorly understood. The success of SCNT in mice gives promise to applications such as species preservation, livestock propagations, and cell therapy for medical treatment^15^,^16^. While cloning mammals by SCNT has been achieved in many species, the success rate is extremely low, with a high incidence of developmental abnormalities^17^. Given that developmental defects of SCNT embryos first appear at the time of zygotic genome activation (ZGA), which occurs at the 2-cell stage in mouse and at the 4- to 8-cell stage in pig, bovine, and human^18^, it has been postulated that SCNT embryos have difficulties in ZGA due to undefined epigenetic barriers preexisting in the genome of donor cells^19^. Although previous studies have identified a number of dysregulated genes in mouse 2-cell SCNT embryos^20^,^21^,^22^, and in the late cleavage stage human SCNT embryos^23^, the nature of the presumed “preexisting epigenetic barriers” and their relationship with impaired ZGA in SCNT embryos remains unknown.

In the present study, we show that autophagy is absent during the activation period in SCNT embryos and influences the maternal mRNA degradation. Mechanistically, the depolymerized actin filament causes the absence of autophagy in SCNT embryos and chemical modulator of autophagy, rapamycin and pp242, can restore the autophagy during activation period and effectively improve the development of cloned mouse embryos.

## Materials and Methods

### Animals

B6D2F1 (C57BL/6 X DBA/2) female/male mice were obtained at 8–12 weeks of age from Beijing Vital River Laboratory Animal Technology Co. Ltd. They were housed in rodent breeding cages at 22 °C ± 1 °C with 70% humidity under a 14/10 h light–dark regime. food and water were available ad libitum. All animal care and experiments were carried out in accordance with the Guidelines for Animal Experiments of the Harbin Medical University. The protocol was approved by the Committee on the Ethics of Animal Experiments of the Harbin Medical University.

### Oocyte collection and spermatozoa preparation

Female B6D2F1 mice were superovulated by intraperitoneal injection of 5 IU pregnant mare serum gonadotropin (PMSG, NSH, China), followed 48 h later by 5 IU human chorionic gonadotropin (hCG, NSH, China). Oocytes were collected from the oviducts 14 h after hCG injection. After collection, cumulus cells were removed from oocytes with 300 μg/ml hyaluronidase (Sigma, H4272) in droplets of HEPES-CZB by gentle pipetting, and then washed in HEPES buffered CZB medium (HEPES-CZB) for several times and resuspended in HEPES-CZB containing 3%PVP (Sigma, PVP360) and used as donor cells for SCNT directly. Denuded oocytes with homogeneous ooplasm were selected and kept in new droplets of CZB medium containing 5.6 mM glucose (CZBG), covered with sterile mineral oil (Fisher, O121–20), then incubated at 37 °C in a 5% CO_2_ atmosphere until use. Spermatozoa were collected from the cauda epididymis of 8–12 weeks age of B6D2F1 males, then kept in CZB-HEPES medium and prepared for injection.

### Generation of ICSI, NT and PA embryos

ICSI was carried out by a piezo-driven unit using the methods as described elsewhere^24^, except that our experiment was performed in HEPES-CZB containing 5 μg/ml cytochalasin B (CB; Sigma, C6762) at room temperature^25^. Only the sperm head was injected into oocyte. After 10–20 min recovery, the ICSI-generated embryos were washed for several times and cultured in KSOM at 37 °C in a 5% CO_2_ atmosphere. In order to eliminate any possible effects brought by NT methods to following investigation, we adopted the same one-step micromanipulation technique to reconstruct SCNT embryos as described previously^26^ with modifications. Briefly, the cumulus cell’s membrane was broken with several piezo pulses then 5–10 cells were sucked into the injection pipette. Oocyte M II spindle was adjusted to 8–10 o’clock then one cumulus cell was injected into the nearby plasma. The spindle was sucked into the injection pipette immediately and taken out of oocyte. One hour after NT, the reconstructed SCNT embryos were activated with 5 mM SrCl_2_ (Sigma, 439665) in Ca^2+^-free CZB containing 5 μg/ml CB (activation medium) for 6 h. For parthenogenetic activation (PA), collected M II oocytes were directly activated with 5 mM SrCl_2_ (Sigma, 439665) in activation medium for 6 h. After activation, the embryos were washed in KSOM and cultured in the same condition as ICSI embryos.

### Treatment of chemical modulator of autophagy and observation of preimplantation development

Rapamycin (Sigma) and PP242 (Sigma) were dissolved in DMSO. To obtain embryos of homogenous quality, NT embryos were selected at 1 hr after donor cell injection. The embryos were collected and washed three times in HEPES-CZB medium, and then randomly cultured in the activation medium containing 10 nM rapamycin or 100 nM pp242 for 6 h. Embryos in the control group were incubated with the same amount of solvent (DMSO). The percentages of blastocyst stage embryos were counted under a dissecting microscope at 120–122 hr after the culture of the zygotes. Experiment was repeated at least ten times. Images of treated embryos were captured using phase contrast microscopy (Olympus America Inc., Center Valley, PA).

### RNA extraction, reverse transcription and quantitative polymerase chain reaction (qPCR)

M II oocytes, embryos at 3 h, 6 h, 9 h, 22 h, 48 h, 60 h, 72 h, 96 h and 120 h after fertilization or activation were collected as at pronucleus, 2-cell, 4-cell, 8-cell, morula, early blastocyst and late blastocyst stage, respectively. Each embryo sample was added with 105 copies of XenoTM RNA from SYBR Green Cell-to-CTTM Control Kit (Life Technologies, 4402959) during cell lysis as external control. Frozen–thawed embryos were used to quantify mRNA levels. Poly (A) mRNA from pools of 10 embryos was isolated from embryos by using the Dynabeads mRNA DIRECT Micro Kit (Invitrogen). Due to the low concentration of extracted mRNA, integrity, and purity were not verified^27^. Oligo (dT) 25-coupled Dynabeads and mRNA complexes were immediately used for reverse transcription using the High Capacity cDNA Reverse Transcription Kit (ABI, 4368814), according to the manufacturer’s instructions. Real-time quantitative PCR reactions were performed at 50 °C for 2 min and 95 °C for 10 min followed by 40 cycles at 95 °C for 15 s, 60 °C for 15 s and 72 °C for 15 s using Top Green qPCR SuperMix (TransGen, AQ131) on CFX96 Real-Time System (Bio-Rad, USA). Real time RT-PCR was performed using the 7 primer sets listed in Table S1. The cDNA levels of target genes were analyzed using comparative Ct methods and normalized to candidate references, *H2afz, Hprt1* and ExnoTM were used as candidate references for embryos^28^.

### Immunofluorescent detection and microscopy analyses

M II oocytes and Embryos at 3 h, 6 h and 9 h post donor cell/sperm injection (hpa) were collected. The zona pellucida was removed by using acidic tyrode solution. After washing in HEPES-CZB, the oocytes or embryos were fixed in 4% paraformaldehyde (Sigma, PFA, P6148) in phosphate-buffered saline (PBS) at room temperature for 40 min. Embryos were permeabilized in 0.2% Triton-X 100 in PBS at room temperature for 1 h. For detection of 5 mC and 5 hmC, permeabilized embryos were additionally incubated in 4 N HCl solutions at room temperature for 10 min followed by neutralization in Tris-HCl, pH 8.0, for 10 min. After permeabilization, the embryos were blocked overnight at 4 °C in 1% BSA, 0.2% Triton X-100 in PBS. Embryos were incubated with anti-5 hmC (rabbit polyclonal; Active Motif) and anti-5 mC antibodies (mouse monoclonal; Calbiochem) in blocking solution for 1 h at room temperature, or incubated with anti-LC3 (rabbit monoclonal; Proteintech) and anti-p62 (mouse polyclonal; Proteintech) in blocking solution for overnight at 4 °C. The embryos were washed several times in 0.01% Tween 20 in PBS (PBST), transferred to secondary antibody mixture of Alexa Fluor 568 goat anti-mouse and Alexa Fluor 488 goat anti-rabbit (Invitrogen), and incubated at room temperature for 1 h. After the nuclei were stained with 10 μg/ml Hoechst 33342, the embryos were mounted on slides with DABCO (Beyotime, P0126), and observed with laser-scanning confocal microscope (Zeiss, LSM700). Quantitative analysis of LC3 dots and pronuclei was done using Image-pro plus version 6.3 (Media Cybernetics Inc.). All software settings for intensity and saturation were maintained constant across all experimental groups.

### Western blotting

150–200 embryos were collected in SDS sample buffer (10 mmol/L, pH 6.8, Tris–Cl, 20 mmol/L DTT, 4% SDS, 0.2% bromophenol blue, and 20% glycerol), respectively, and heated to 100 °C for 5 min. The total proteins were separated by SDS–PAGE with a 5% stacking gel and 12% separating gel at 60 V, 0.5 h and 100 V, 2 h, respectively, and then electrophoretically transferred to nitrocellulose membrane (Bio-Rad Laboratories, Hercules, CA, USA) for 1.5 h, 0.65 mA/cm2. Following transfer, blocking in 5% skimmed milk in TBST (TBS containing 0.1% Tween 20) at 4 °C overnight, the membrane was incubated in TBST containing 1:1000 anti-LC3 (rabbit monoclonal; Proteintech) or GAPDH antibody(Cell Signaling technology) at 37 °C for 2 h. The membrane was then incubated with horseradish peroxidase-conjugated secondary antibodies (Santa Cruz Biotechnology, Santa Cruz, CA, USA) diluted 1:1000 in TBST at 37 °C for 1 h. The signals were visualized by the DAB detection system.

### Statistical Analysis

The general linear models (GLM) procedure in the Statistical Analysis System (SAS User’s Guide, 1985, Statistical Analysis System Inc., Cary, NC) was used to analyze the data from all the experiments. Significant differences were determined using Tukey’s multiple range test and P < 0.05 was considered significant.

## Results

### Expression of LC3 in mouse ICSI, SCNT and PA embryos

**Figure 1:**
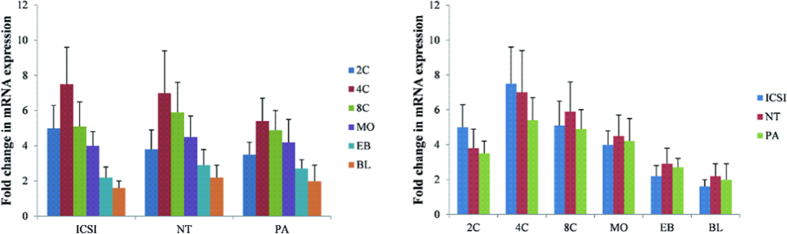
Temporal expression patterns of the *Lc3* mRNA. Relative mRNA expression levels of *Lc3* at different developmental stages in ICSI, NT and PA analyzed by qRT PCR. mRNA expression at the meiosis II (M II) stage was arbitrarily set as onefold. Fold differences in the mRNA expression from equivalent numbers of two-cell (2C), four-cell (4C), eight-cell (8C), morula (MO), early blastocyst (EB) and blastocyst (BL) stage embryos are shown after normalisation against the internal standard *H2afz* and *Hprt1*. Data are presented as the mean ± s.e.m. of three separate experiments.

To investigate the expression pattern of autophagy in SCNT embryos, we firstly examined the quantity of *Lc3* (*Atg8*) mRNA in mouse SCNT preimplantation embryos compared with the ICSI and PA counterparts from the 2-cell to the blastocyst stage. All the three types of embryos showed similar pattern of *Lc3* mRNA expression, which was highest at the 4-cell stage and gradually decreased from the 8-cell to the blastocyst stage (Fig. 1a). Furthermore, there was no significant difference among 3 types of embryos at individual developmental stages (Fig. 1b). These results indicate that the SCNT embryos have normal pattern of autophagy after ZGA.

**Figure 2:**
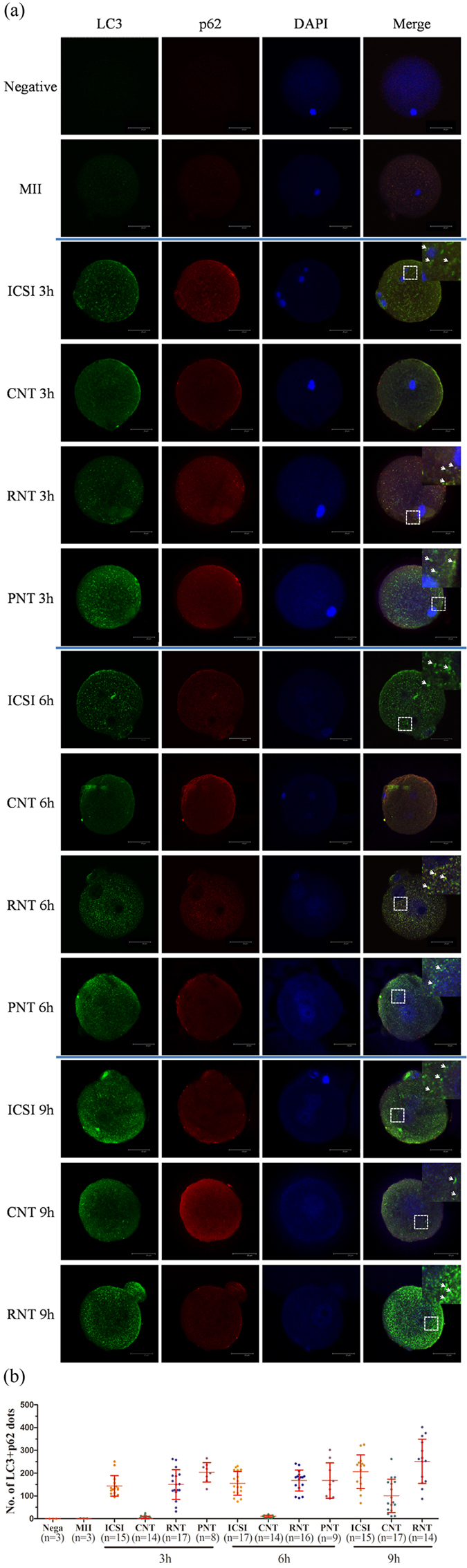
Autophagy in pronucleus stage formation. (**a**) Coexpression of LC3 and p62 in the meiosis II (M II), pronucleus stage of ICSI, NT (CNT), rapamycin treated NT (RNT), and pp242 treated NT(PNT). Green, LC3 labelled protein; Red, p62 labelled protein; Blue, chromatin; Merge, LC3 (green), p62 (red) and chromatin (blue) merged images. Note that the colocalisation of LC3 and p62 is indicated by an arrow in the enlarged panel, which is indicated by the white box. Scale bar, 20 μm. (**b**) Quantification of LC3 and p62 merged dots in oocytes and embryos. Each value represents the mean ± s.e.m. *p < 0.05; **p < 0.01; ns, not significant.

Next, we examined the LC3 protein expression in pronucleus stage of SCNT embryos compared with ICSI and PA counterparts by immunofluorescence. It is known that conversion of cytosolic LC3 (LC3-I) to membrane bound phosphatidylethanolamine (PE)–conjugated LC3 (LC3-II) occurs during autophagy induction, and that the amount of LC3-II correlates with the number of autophagosomes^29^. Likewise, the number of LC3-labeled dots represents autophagic vesicles in different types of cells. P62 (also known as SQSTM1), could colocalize with LC3 through direct binding^30^. We double stained the p62 protein with LC3 to ensure the authenticity of the experiment and the number of autophagosomes (punctate structures of LC3 and p62) in each oocyte or embryo was analyzed. Although M II oocytes rarely showed LC3 and p62 signals, a lot of dots representing autophagosomes appeared in ICSI embryos at the 3 h, 6 h and 9 h pronuclear stage (Fig. 2). By contrast, in SCNT, the 3 h and 6 h embryos showed few dots of LC3 and p62; only 9 h group appeared the dots with significant small amount. Interestingly, PA counterparts also showed similarly phenomenon as SCNT (Fig. S1). LC3 dots were not observed in 6 h embryos; in contrast, LC3 dots were generated in embryos from 9 h group. These results suggest that the autophagy is not triggered in SCNT embryos during 6 hours of activation.

### Treatment of mTORC1 inhibitors induces autophagy during activation of SCNT embryos

**Figure 3:**
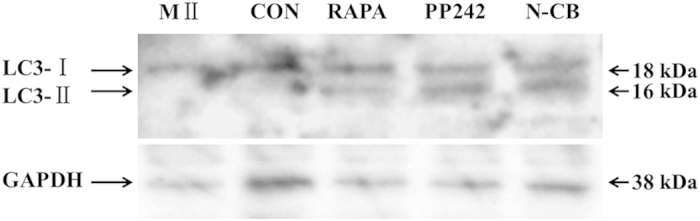
Western blotting assay for conversion of LC3-I (cytosolic) to LC3-II (autophagosome bound). LC3-I (lane 1), LC3-II (lane 2), and GAPDH (lane 3) in M II oocytes and 6 h after activation of NT (CON), rapamycin treated NT (RAPA), pp242 treated NT embryos (PP242) and non CB treated embryos (N-CB). Full-length blots are presented in Supplementary Fig. 3.

Rapamycin and PP242 are specific inhibitor of mTORC1 (mammalian target of rapamycin), which can induce autophagy in mammalian cells^31^,^32^,^33^. Since there were differences of autophagy expression between SCNT and ICSI embryos within 6 hours after activation or sperm injection, we then treated SCNT embryos with autophagy inducers in order to address whether the artificial adjustment can recruit the autophagy during activation period in SCNT embryos. Immunofluorescent staining method was used for analysis the number of autophagosomes. The number of LC3 and p62 puncta formation was very low in 3 h, 6 h group, and only detected in 9 h group in the normal NT groups; however, when SCNT embryos were incubated in activation media in the presence of rapamycin or pp242, the LC3 combined p62 dots were increased in 3 h and 6 h groups (Fig. 2). Additionally, induction of autophagy was also confirmed by LC3 conversion. Western blot analysis showed that the amount of LC3 was also more than control group in the rapamycin-treated or pp242-treated embryos (Fig. 3). These data suggest that the treatment of autophagy inducers during activation period of SCNT embryos can restore the expected level of autophagy.

### Activation of autophagy greatly improves development of SCNT embryos

**Figure 4:**
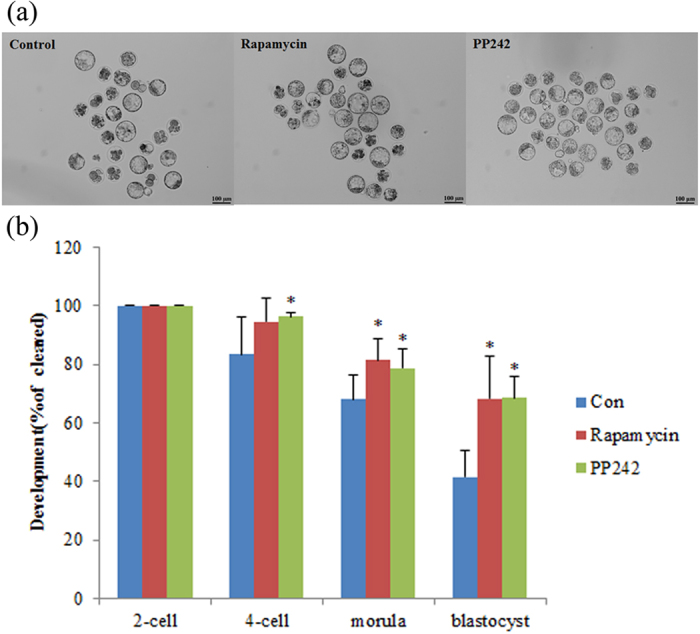
Activation of autophagy greatly improves development of SCNT embryos. 10 nM of rapamycin or 100 nM of pp242 was added into activation medium for 6 h during the activation of NT embryos. (**a**) The embryonic stages at 22 h, 46 h, 72 h and 96 h after activation were calculated as mean ± SD. *p < 0.05. (**b**) Representative bright-field photographs of embryos at blastocyst stage are shown. Scale bar, 100 μm.

To examine the effect of restored autophagy level of SCNT embryos following rapamycin or pp242 treatment, we first analyzed the developmental potential of SCNT embryos. In control SCNT embryos, the developmental rate began to decline after the first cleavage with 41.5% of cleaved embryos successfully developing to the blastocyst stage after 96 hpa (Fig. 4, Table S2), a finding consistent with previous studies^34^. Strikingly, SCNT embryos treated with rapamycin or pp242 rarely arrested during 2- to 4-cell and 4-cell to morula stage transition and developed to the blastocyst stage with high efficiency (68.5% and 68.7%; Fig. 4; Table S2). These data indicating the improvement of SCNT embryo development depends on the restored autophagy level by treatment of autophagy inducer.

**Figure 5:**
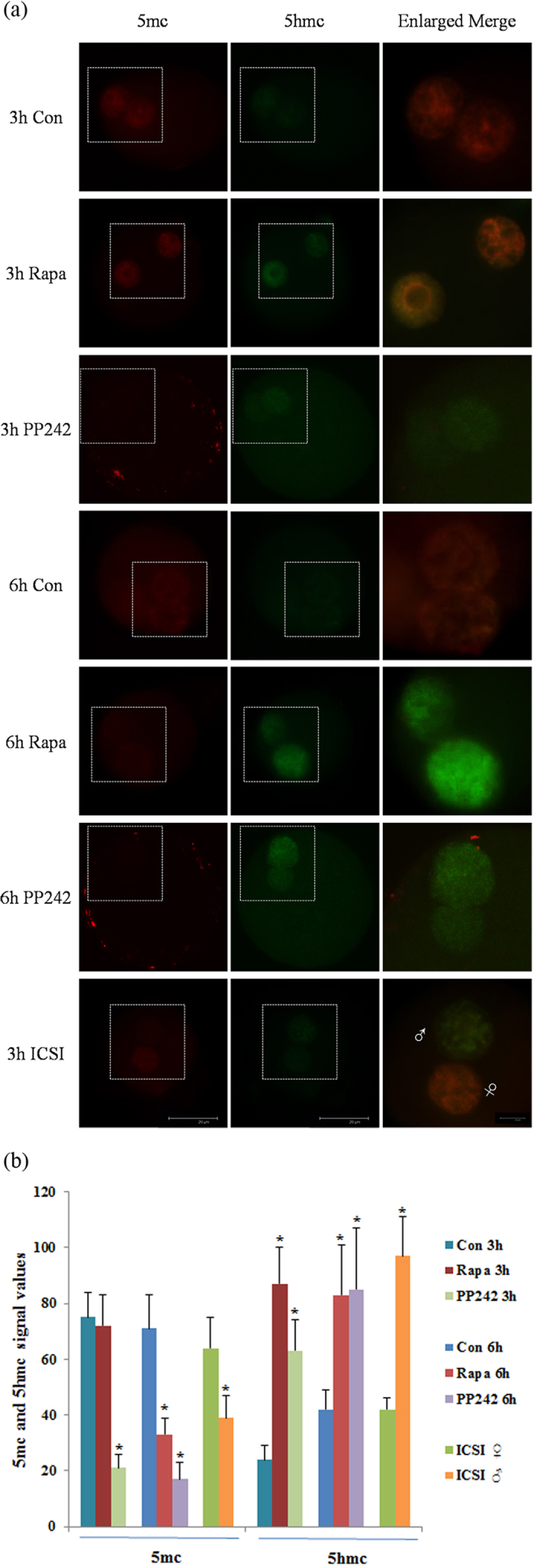
Expression of 5 hmC and 5 mC signals during activation period. (**a**) Immunofluorescent images of 5 mC (red) and 5 hmC (green) staining, and merged images in 3 h and 6 h after activation of NT (Con), rapamycin treated NT (Rapa), pp242 treated NT embryos and 3 h after sperm injection of ICSI embryos. White boxes indicate the pronuclear area; ♂, male pronucleus; ♀, female pronucleus; Scale bar, 20 μm. (**b**) Semi-quantitative analysis of fluorescence intensity for 5 mC and 5 hmC in pronuclear of embryos. Each value represents the mean ± s.e.m. of at least 10 oocytes or embryos from the indicated stages. *p < 0.05.

The conversion of 5-methylcytosine (5 mC) into 5-hydroxymethylcytosine (5 hmC) is an intermediate process of the active DNA demethylation in animals. Therefore, we analyzed the DNA demethylation status by observing the 5 mC and 5 hmC expression during activation period. The conversion of 5 mC to 5 hmC in embryo was demonstrated by immunofluorescence staining. In ICSI embryos, an asymmetric level of 5 mC and 5 hmC was observed in paternal and maternal pronuclei that the paternal pronucleus possessed a low level of 5 mC and high level of 5 hmC compared to the maternal pronucleus (Fig. 5). Interestingly, we found both the 5 mC and 5 hmC showed similar level between pseudo-pronuclei of SCNT embryos. In addition, the 5 mC signal was enriched in SCNT embryos whereas there was very weak 5 hmC signal, indicating insufficient reprogramming. In contrast to the untreated SCNT embryos, 5 hmC were highly expressed in SCNT embryos with rapamycin or pp242 treatment, yet little 5 mC signals were detected (Fig. 5). This situation can be observed both in pseudo-pronuclei at 3 h and 6 h. The observed decrease in 5 mC and increase in 5 hmC levels in the rapamycin or pp242 treated pseudo-pronuclei indicate that the accelerative conversion of 5 mC to 5 hmC causes active DNA demethylation.

### Effect of rapamycin treatment on maternal mRNA degradation

**Figure 6:**
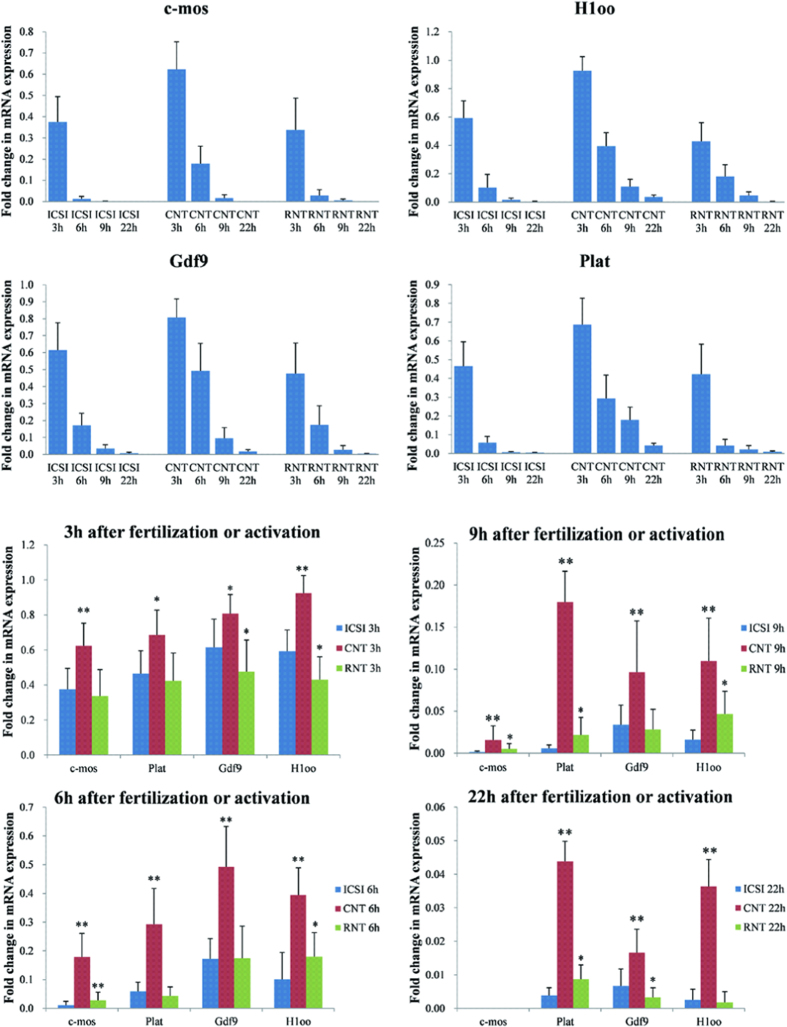
Degradation of selective maternal mRNA after fertilization or activation. mRNA was isolated from embryos 3 h, 6 h, 9 h and 22 h after sperm injection or activation, Reverse-transcribed using oligo(dT) primer, and subjected to qPCR to examine the changes in the relative amount of *c-mos, Plat, Gdf9 and H1oo* mRNAs. The values of the M II oocytes were set as onefold. Three independent experiments were performed and averaged values are shown. *p < 0.05; **p < 0.01.

We analyzed the function of autophagy in the removal of obsolete maternal factors. To address this issue, we compared four typical maternal mRNA level in rapamycin treated SCNT embryos with control SCNT and ICSI embryos. We conducted qRT-PCR in M II oocyte and embryos at 3 h, 6 h, 9 h and 22 h (2-cell stage) after activation/sperm injection. The amount of *c-mos, Plat, Gdf9* and *H1oo* mRNAs decreased dramatically by 9 h and reached marginal or undetectable levels at 22 h in either SCNT or ICSI embryos (Fig. 6a), indicating that these maternal mRNA specifically undergo rapid degradation. However, when we compared the ICSI and SCNT embryos in each time intervals, the mRNA expression of *Plat, Gdf9 and H1oo* was significantly higher in 3 h, 6 h, 9 h and 22 h of SCNT embryos than that of ICSI embryos counterparts (Fig. 6b). The status of *c-mos* mRNA expression also showed similar status beside undetectable at 22 h. After rapamycin treatment, the amount of all of the examined mRNA was decreased at each interval compared with the untreated SCNT embryos, and close to ICSI counterparts (Fig. 6b). These results suggest that the rapid degradation of maternal mRNA is, at least in part, triggered by autophagy. In addition, the expression of maternal mRNA in PA embryos during pseudo-pronucleus stage was also higher than that of the ICSI embryos and decreased after rapamycin treatment (Fig. S2). On account of the same method of activation in SCNT and PA embryos, we believe that the differences of autophagy and maternal mRNA expression during activation period between ICSI and SCNT embryos are due to the different activation methods.

### Depolymerization of actin filaments affects autophagy induction during SCNT embryos activation

**Figure 7:**
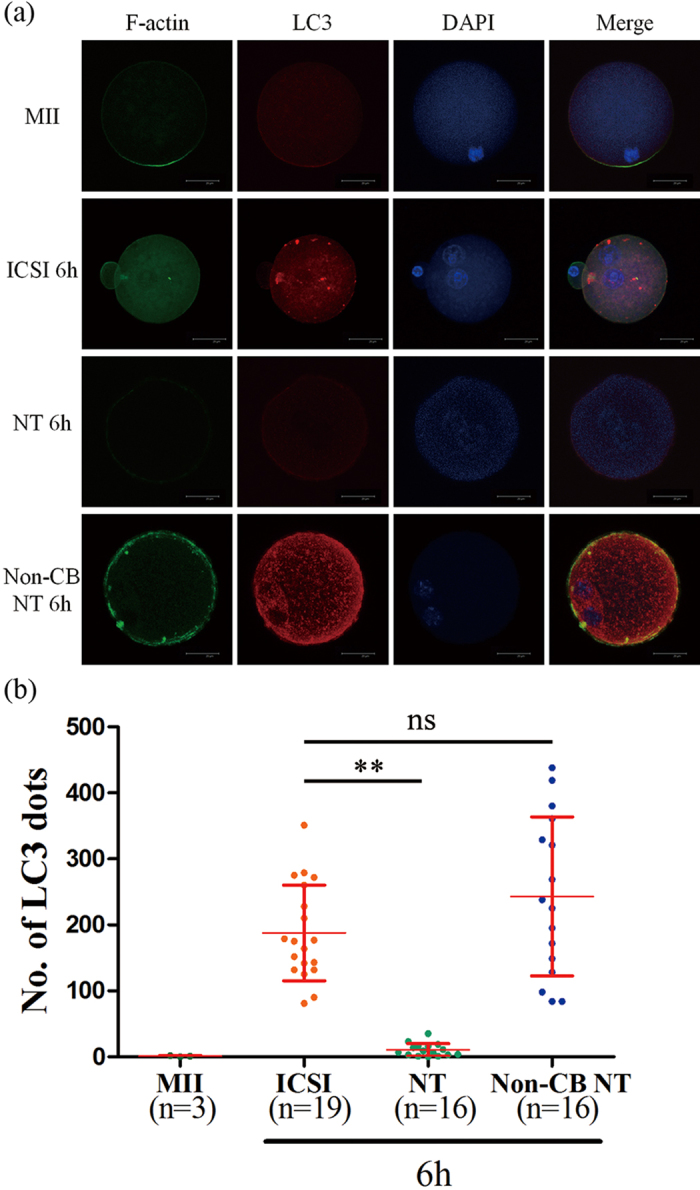
Double staining of F-actin and LC3. (**a**) M II oocytes, ICSI embryos 6 h after sperm injection, NT embryos 6 h after activation with or without CB in activation medium were stained by phalloidin (green) and LC3 antibody (red). Nuclei were visualized by DAPI. Scale bar, 20 μm. (**b**) Quantification of LC3 dots in oocytes and embryos. Each value represents the mean ± s.e.m. *p < 0.05; **p < 0.01; ns, not significant.

In ICSI embryos, the direct injection of sperm into the cytoplasm can induce activation of oocytes. By contrast, SCNT and PA embryos must be activated manually by electrical pulse or SrCl_2_ treatment in order to mimic the calcium oscillation of fertilization. CB was used as actin filament inhibitors for the diploid complement retention of SCNT and PA embryos. In order to address whether the inhibition of actin filament participates in the autophagic pathway during SCNT embryo activation, we removed the CB from the activation medium. After double staining of F-actin and LC3, in M II oocytes, microfilaments were found mainly in the cortex but with a great concentration in the area near the spindle chromosome complex. In ICSI embryos, distinct microfilaments were detected in cortical region of the cytoplasm with a mass of LC3 dots in cytoplasm. Compared with ICSI embryos, both of microfilaments and LC3 dots were disappeared in SCNT embryos. However, a distinct microfilaments and lots of LC3 dots were observed in non-CB treated SCNT embryos (Fig. 7). Western blot analysis showed that the expression level of LC3 in non-CB treatment embryos was increased and similar with rapamycin-treated or pp242-treated group (Fig. 3). These results suggest that polymerization of actin filament is necessary at certain steps during the autophagic process induced by SCNT activation. Furthermore, the decrease autophagic induction during SCNT activation is caused by depolymerization of actin filaments.

## Discussion

A landmark study demonstrated a critical role of autophagy in early mammalian preimplantation development at the stage of transition from the maternal to zygotic gene program^4^. Autophagy has also been shown to participate in the regulation of the somatic reprogramming process in iPSCs^12^. In current study, we first demonstrated the presence of LC3 mRNA in mouse SCNT preimplantation embryos using quantitative real-time RT-PCR and immunocytochemistry. LC3 is first isolated as a microtubule-associated protein and subsequently localizes to autophagosomes and isolation membranes during autophagy^29^. It specifically associates with growing phagophores and mature autophagosomes^35^. This association is essential for the extension, curvature and closure of the isolation membrane to form the autophagosome^5^. We found that LC3 mRNA expression level were similar between SCNT and ICSI embryos from 2-cell to blastocyst stages. But during the pseudo-pronucleus stage, the autophagosome formation represented by LC3 together with p62 was nearly not induced at first 6 h of activation in SCNT embryos. The same phenomenon could be observed in PA embryos as well. The SCNT and PA embryos are activated by SrCl_2_ in order to mimic the calcium oscillation of fertilization and CB is added to maintain diploid karyotype, while ICSI embryos are directly activated by sperm injection into cytoplasm. CB is generally used as actin filament inhibitor to preventing the extrusion of the second polar body of reconstructed SCNT or PA embryos. Several studies have shown that the actin cytoskeleton participates in the formation of autophagosomes. Accumulating evidence suggests that microfilaments are essential for selective types of autophagy in yeast^36^,^37^,^38^. In mammals, only a little report about the relationship between the actin cytoskeleton and autophagy has been published. Recently, a report shows that actin depolymerization is affects very early steps of autophagosome formation^39^. In another report it has been shown that the depolymerization of actin using Cytochalasin B or D decreases the degradation of long-lived proteins and also prevents the accumulation of autophagic related structures. Our findings about the non-CB treated SCNT embryos showing lots of LC3 dots during activation period combined with distinct microfilaments presence are consistent with these previous observations, and expand those results indicating polymerization of actin filament is necessary at certain steps during the autophagic process induced by activation/sperm injection in mouse embryos. Moreover, the inhibited autophagic induction during SCNT activation is caused by depolymerization of actin filaments. Our observations seem to be inconsistent with previous study that parthenogenetic activation also induces autophagy^4^. In their study, oocytes isolated from GFP-LC3 mice were parthenogenetically activated with SrCl_2_ and CB for 4 h. In these oocytes, GFP-LC3 dot generation was observed 6 h after activation. However, they only activated the PA embryos 4 hr compared with 6 hr of our protocol. In our results, we can observe the autophagosomes were emerged in 9 hpa SCNT embryos, indicating that the microfilaments and autophagy will restore after activation. Thus, our result is consistent with the previous study beside the period of activation.

Rapamycin and PP242, a specific inhibitor of mTORC1, induces autophagy in mammalian cells as well as in Saccharomyces cerevisiae and Drosophila melanogaster^31^,^32^,^33^,^40^,^41^,^42^. In order to rescue the insufficient autophagy in SCNT embryos, we treated the SCNT embryos with rapamycin or pp242 during the 6 hours of activation, which restored the expected level of autophagy. In contrast, it is previously reported that treatment of CHO and HeLa cells with the actin depolymerizing agent Latrunculin B or Cytochalasin B abolished the rapamycin- and starvation-dependent increase of LC3-positive dots and the LC3-II levels, without affecting basal autophagy^39^. In addition, Yamamoto *et al.* reported that fertilization-induced autophagy in mouse embryos was independent of mTORC1^43^. Therefore, as our results, autophagy was induced by rapamycin or pp242 in SCNT embryos during activation period even the presence of CB in activation media. It is likely that further downstream factors are shared by fertilization-induced autophagy and canonical autophagy.

We next examined whether the restoration of autophagy during activation period can improve the cloning efficiency in SCNT embryos. In the present study, we treated SCNT embryos with 10 nM rapamycin or 100 nM pp242 following oocyte activation, which resulted in more efficient *in vitro* development of SCNT embryos to the blastocyst stage. Moreover, the conversion of 5 mC to 5 hmC in SCNT embryos was accelerated after rapamycin or pp242 treatment. DNA methylation at the 5-position of cytosine (5-methylcytosine; 5 mC) is one of the key epigenetic marks that play a crucial role in development and genome regulation^44^,^45^,^46^,^47^. 5 hmC serves as an intermediate between 5 mC and unmethylated C. Thus, 5 hmC is an intermediate during the active DNA demethylation process in animals. Recently, it was shown that 5 hmC existed in mouse, bovine and rabbit zygotes, and that 5 hmC accumulated specifically in the paternal pronucleus coinciding with a reduction in 5 mC^48^,^49^, which suggests a potential biological function of 5 hmC and a role in the regulation of DNA methylation dynamics in early development. A report showed that reprogramming in SCNT might thus share a common mechanism with paternal genome remodelling in fertilized eggs^50^. However, aberrant reprogramming of 5 mC and 5 hmC was observed in porcine SCNT embryos during early development^51^. Although lack of systematic study about the reprogramming of 5 mC and 5 hmC in mouse SCNT embryos during early development, our results clearly showed that the conversion of 5 mC to 5 hmC in SCNT embryos was blocked. After rapamycin or pp242 treatment, the conversion of 5 mC to 5 hmC in SCNT embryos was accelerated and it was considered one of reasons for improved cloning efficiency after induction of autophagy in SCNT embryos.

We have demonstrated that rapamycin or pp242 is an effective and efficient method to restore the autophagic level which is suppressed by CB during activation in SCNT embryos. Furthermore, the restoration of autophagy significantly improves cloning efficiency via accelerating the conversion of 5 mC to 5 hmC. In addition, the conversion of 5 mC to 5 hmC depends on the presence of ATP^52^. During oogenesis, oocytes accumulate maternal proteins necessary for oogenesis. Although many of these proteins are provided to zygotes (fertilized embryos) maternally, the stockpile is largely degraded after fertilization, and newly synthesized proteins encoded by the zygotic genome are translated. This process, well known as the oocyte-to-embryo transition, occurs concurrently with many other changes, including maternal RNA degradation, RNA synthesis, and organelle remodeling. In mice, zygotic transcripts are detected at the late one-cell stage, and most of the maternal RNAs are eliminated by the two-cell stage^53^. Recent studies suggested that micro RNAs participated in the degradation of maternal mRNAs^54^,^55^,^56^. However, it remains to be resolved how maternal cytoplasmic contents are degraded after fertilization. We hypothesize that high level of autophagy induction during pronucleus stage is important for exchanging the maternal cytoplasmic contents into zygotic ones, that is, to generate maternal protein-derived amino acids, which could be utilized for new protein synthesis during preimplantation development. By examination of the four rapidly degrading mRNAs (*c-mos, Plat, Gdf9 and H1oo*)^3^ in pronucleus stage in this study, the qPCR data showed significantly retardatived maternal mRNA elimination in SCNT embryos. After rapamycin treatment, the amount of all of the mRNA examined decreased at each interval compared with the control SCNT embryos, and close to ICSI counterparts. These results suggest that the rapid degradation of maternal mRNA is, at least in part, triggered by autophagy.

In conclusion, we have demonstrated that depolymerization of actin filaments by CB treatment during the activation period suppressed the expression of autophagy in SCNT embryos. Furthermore, the recruitment of autophagy by rapamycin or pp242 could dramatically improve cloning efficiency. Our findings suggest that this initial induction of autophagy plays an important role in reprogramming and is important for the further development of cloned embryos.

## Additional Information

**How to cite this article**: Shen, X.H. *et al.* Induction of autophagy improves embryo viability in cloned mouse embryos. *Sci. Rep.* **5**, 17829; doi: 10.1038/srep17829 (2015).

## Supplementary Material

Supplementary Information
