# Supplementary material for: Induction of autophagy improves embryo viability in cloned mouse embryos
Source: Sci Rep. 2015 Dec 8;5:17829. doi: 10.1038/srep17829 (PMC4672298; doi:10.1038/srep17829)

Supplemental Material to: Induction of autophagy improves embryo viability in cloned mouse embryos

XingHui Shen, [1†] Na Zhang, [1†] ZhenDong Wang, [1] GuangYu Bai, [1] Zhong Zheng, [1] YanLi Gu, [1] YanShuang Wu, [1] Hui Liu, [1] DongJie Zhou[1] and Lei Lei[1 *]

†The authors consider that the first two authors should be regarded as joint first authors.

*Correspondence to: Lei Lei; Email: Leiys2002@yahoo.com

Supplementary Table 1. List of Primers used in this work.

**Gene**	**Accession No.**	**Primer sequence （5'-3'）**	**Product size**	**Efficiency**	**R^2**
H2afz	NM_016750	F: CTGAAGTAGTGGGTTTTGATTG<br>R: GGGATATGACCTTTATTGAGCT	147	99.0%	0.998
Hprt1	NM_013556	F: CAGCGTCGTGATTAGCG<br>R: GCCTCCCATCTCCTTCAT	160	97.3%	0.999
Lc3	NM_026160	F: TTCTTCCTCCTGGTGAATGG<br>R: GTGGGTGCCTACGTTCTCAT	216	97.2%	0.991
c-mos	NM_020021	F: CGCCACGACAACATAGTTCG<br>R: ACCGTAGATGACTTGGTGTAGAG	123	95.7 %	0.998
Plat	NM_008872	F: AACGCAGACAACTTACCAACA<br>R: GTTCGCTGCAACTTCGGAC	131	104.6%	0.995
Gdf9	NM_008110	F: GTCACCTCTACAATACCGTCCG<br>R: CACCCGGTCCAGGTTAAACA	119	100.0 %	0.999
H1oo	NM_138311	F: AGTTGGCAAGGCCACGATG<br>R: TGACCTCTTTCGGTTTCGCC	110	98.0%	0.999

F, forward; R, reverse.

Supplementary Table 2. The effect of rapamycin in activation medium on SCNT embryo developmental rate.

Development rate of SCNT embryo treated with rapamycin and pp242.

	N	2-Cell (%)	4-Cell (%)	Morula (%)	Blastocyst (%)
Control	681	100	83.5±12.9	68.1±8.4	41.5±9.2
Rapamycin	686	100	94.6±8.3	81.5±7.5*	68.5±14.3*
PP242	167	100	96.3±1.3*	78.9±6.3*	68.7±7.4*

Values were calculated as mean ± SD. *p < 0.05.

Supplementary Figure 1. Double staining of F-actin and LC3. (A) PA embryos were stained by phalloidin (green) and LC3 anti body (red) 6h or 9h after activation. Nuclei were visualized by DAPI. RPA, rapamycin treated PA; Non-CB PA, activate without CB. Scale bar, 20μm. (B) Quantification of LC3 dots in PA embryos. Each value represents the mean ±s.e.m. *p < 0.05; **p <0.01; ns, not significant

Supplementary Figure 2. Degradation of selected maternal mRNA after activation in PA embryos. mRNA was isolated from embryos 3 h, 6 h, 9 h and 22 h after activation, Reverse-transcribed using oligo (dT) primer, and subjected to qPCR to examine the changes in the relative amounts of *c-mos, Plat, Gdf9 and H1oo* mRNAs. The values of the MII oocytes were set as onefold. Three independent experiments were performed and averaged values are shown. *p < 0.05; **p <0.01.

Supplementary Figure 3. Full-length western blotting assay for conversion of LC3-I to LC3-II.

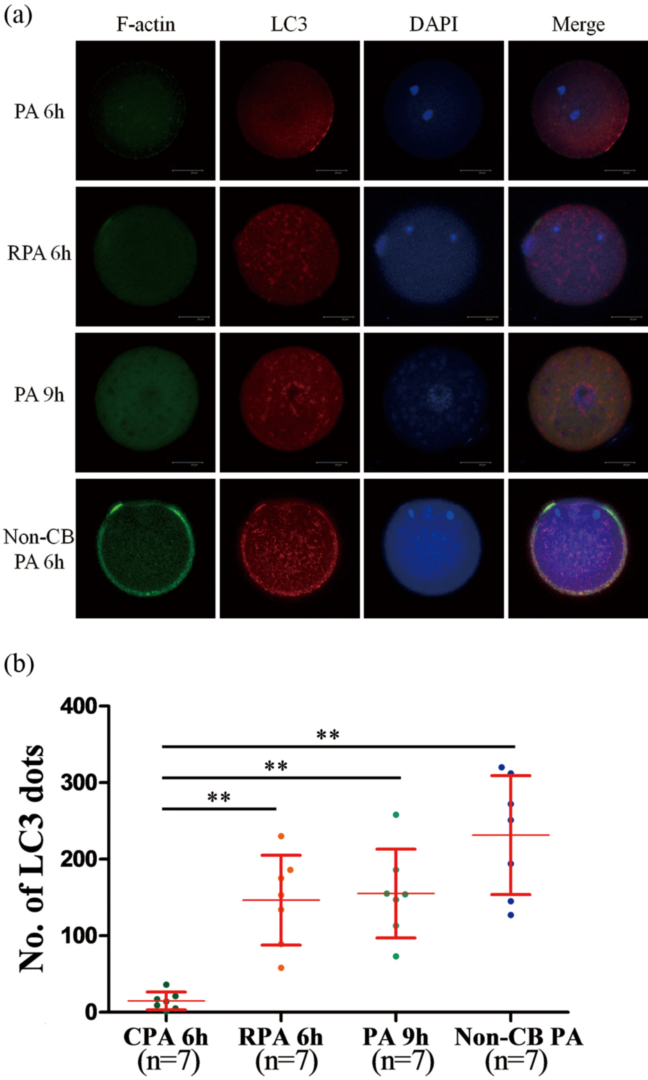
(a)
F-actin
LC3
DAPI
Merge
PA 6h
RPA 6h
PA 9h
Non-CB PA 6h
(b)
No. of LC3 dots
400
300
200
100
0
**
**
**
CPA 6h (n=7)
RPA 6h (n=7)
PA 9h (n=7)
Non-CB PA (n=7)

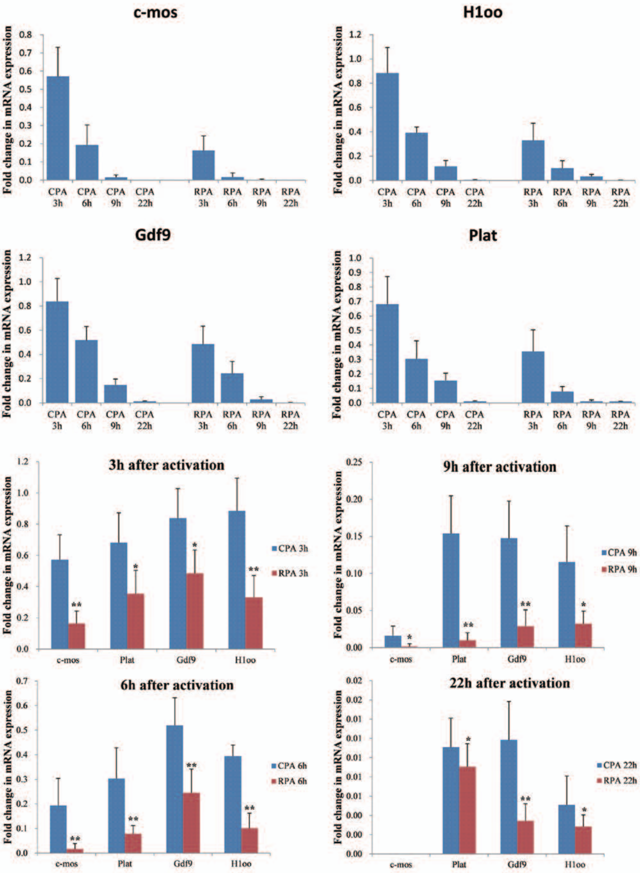
c-mos
H1oo
Gdf9
Plat
Fold change in mRNA expression
CPA 3h
CPA 6h
CPA 9h
CPA 22h
RPA 3h
RPA 6h
RPA 9h
RPA 22h
3h after activation
9h after activation
6h after activation
22h after activation
c-mos
Plat
Gdf9
H1oo
CPA 3h
RPA 3h
CPA 9h
RPA 9h
CPA 6h
RPA 6h
CPA 22h
RPA 22h

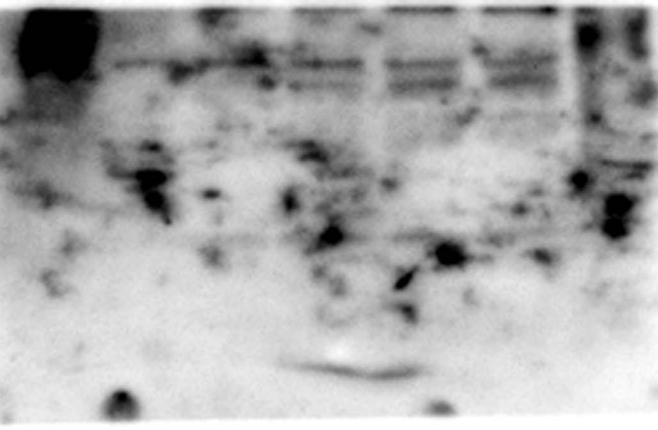

Supplement: Supplementary Information [file srep17829-s1.pdf]
